# Cost-effective wideband dielectric planar lens antenna for millimeter wave applications

**DOI:** 10.1038/s41598-022-07911-z

**Published:** 2022-03-10

**Authors:** José-Manuel Poyanco, Francisco Pizarro, Eva Rajo-Iglesias

**Affiliations:** 1 Department of Signal Theory and Communication, University Carlos III of Madrid, 28911 Madrid, Spain; 2 Pontificia Universidad Católica de Valparaíso, Escuela de Ingeniería Eléctrica, Valparaíso, Chile

**Keywords:** Electrical and electronic engineering, Applied physics

## Abstract

This article presents a fully 3D-printed dielectric planar lens operating in the entire Ka-band manufactured using additive manufacturing and a relatively low-cost 3D-printer. The lens consists of ten concentric rings implemented using low-loss ABS filaments with high permittivity values. By varying the infill percentages of them the required refractive indexes of each section are achieved. An additional 3D-printed matching layer, using the same manufacturing and design method was included in the lens, to reduce reflections. Simulation and measurement results show a very good agreement, which confirms the possibility of manufacturing a cost-effective broadband and planar lens solution operating in millimeter wave bands, where Low Earth Orbit Satellites (LEO) networks, future mobile communication systems (5G, 6G) and radar systems operate.

## Introduction

The new generation of communication systems is migrating to a higher part of the electromagnetic wave spectrum, namely millimeter waves^1^. The main problem with moving up in frequency is that propagation losses increase, making the use of high gain antennas a need to mitigate this drawback^2^. The traditional solutions are based on the use of printed technology as patch arrays, which can achieve high gains but require complex and expensive feeding networks and, also suffer from the dielectric and conductive losses. For this reason, lens antennas have gained ground as a candidate solution in these scenarios^3–9^. Lens antennas present the advantage of not requiring expensive or complex feeding networks, which can be hard to implement for the target frequencies. However, they have the drawback of being difficult to manufacture using standard fabrication methods, due to their complex shapes and the availability of materials to achieve the specific required refractive indexes. In addition, they are electrically large antennas. Therefore, there is a need to find a cost-effective way to design, manufacture and implement these structures, targeting millimeter wave applications.

One technology used in recent years to implement high frequency structures is 3D-printing^10,11^. Thanks to the introduction of low-loss dielectric and conductive filaments, together with the lower cost and higher precision of the new generation of 3D-printers, it has been possible to implement different high frequency topologies that were either difficult or too expensive to manufacture^12,13^. This brings the opportunity to implement cost-effective solutions. Amongst the solutions found in literature, we can find 3D-printed lenses that use graded index lenses^14,15^, Fresnel Zone plates^16^, Luneburg lenses^17,18^ and Gutman lenses^19^ amongst other implementations^20–23^. However, there is still a need to find solutions that are easy-to-print and that fit under a planar lens category. The latter implies a better integration into any communication system as the volume of the structure is reduced. Covering these features is of interest for applications such as Low Earth Orbit (LEO) satellite networks^24^, fifth and sixth generations (5G, 6G) mobile communication systems, and improvements in radar and detection technology^25^.

There are different techniques in literature to implement planar lenses. One way is to compensate the shape of the non-flattened lens using equivalent refractive indexes to compensate the phase to generate the planar wavefront, for example, using transformation optics^26^. Another used method is directly implement a planar structure based on concentric rings with different refractive indexes that focus and compensate the phase of the incident spherical wavefront^14^. For both cases, we need to vary the material properties of the lens to achieve a desired refractive index, implementing what is known as a graded index (GRIN) lens. This kind of topology can be made using 3D-printing by varying the infill percentages inside of the different sections of the lens^19,27,28^.

In this article, a planar dielectric lens antenna operating in the Ka-band is presented, avoiding shape modifications, designed using the field transformation approach presented in^28^. This transformation approach is used to determine the different relative permittivity values of each zone of the lens to transform a spherical wave impinging in one side of the lens, into a plane wave front at its exit.

## Lens design and fabrication

**Figure 1:**
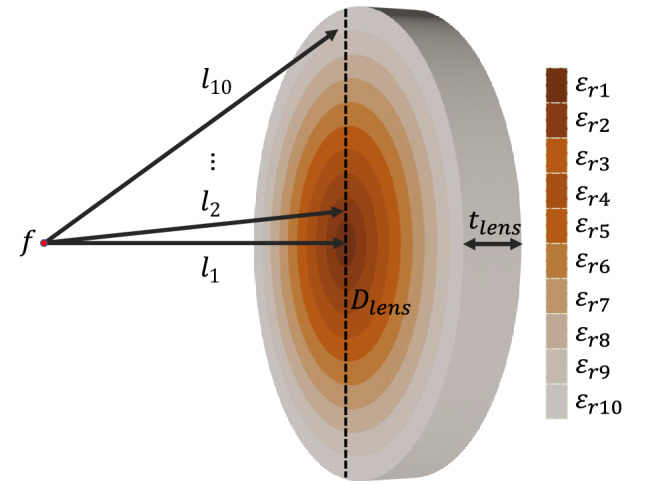
Proposed Graded Index (GRIN) planar lens antenna.

The proposed lens consists of equal-thickness concentric rings of different relative permittivities calculated to give a certain phase delay, with the higher relative permittivity value placed in the center of the lens to achieve a maximum phase delay, and the lower relative permittivity value in the external ring to achieve the lowest phase delay. The structure is depicted in Fig. 1.

The first step for the design of this lens is to define its operational frequency. As we want to operate in the Ka-band, we will set the central operational frequency at 34 GHz. Once the central frequency of the lens is set, we can define the diameter $D_{lens}$ of the lens, its thickness $t_{lens}$ and the corresponding focal distance *f*. For this implementation, we have set $D_{lens}=10\lambda _{0}$, $t_{lens}=D_{lens}/8$, and $f=D_{lens}/3$, which are values within the range of other implementations in literature^14,28^. The last parameter to set is the number *n* of concentric rings. Ideally, this last parameter needs to be set as large as possible to obtain a smooth permittivity gradient in the lens. However, it is necessary to consider the manufacturing method constrains, since the higher the number of rings with a constant lens diameter, the smaller the thickness of each one of them, reaching values that can not be manufactured. For these exposed reasons, the number of concentric rings was set to $n=10$ with a thickness of 4.4 mm each.

The next step in the design of the lens is to obtain the relative permittivity value of each ring. For this purpose, it is necessary to define one relative permittivity for one of the rings, and then calculate the other rings relative permittivity values matching the optical path in the air from the focal point to the surface of each ring ($l_1$,$l_2$,...,$l_{n}$) plus the path inside each ring, to ensure that the waves are in phase when they exit the lens. For this implementation, we define the maximum relative permittivity ($\varepsilon _{r1}$) equals to 12 for the central ring of the structure. Once this value is defined, we calculate the remaining relative permittivities for the concentric rings using Eq. (1)^28^, taking into account its respective optical paths. The calculated values of the relative permittivity of each concentric ring and its corresponding optical lengths are shown in Table 1.

**Table 1:** Optical length and relative permittivity value for each ring.

Ring (*n*)	Optical length ($l_n$) (mm)	Relative permittivity ($\varepsilon _{r_n}$)
1	29.41	12
2	30.15	11.54
3	31.41	10.78
4	33.22	9.73
5	35.49	8.49
6	38.13	7.15
7	41.08	5.79
8	44.27	4.48
9	47.66	3.28
10	51.20	2.22

1 $$\begin{aligned} \varepsilon _n=\left( \frac{(l_{1}-l_{n}+\sqrt{\varepsilon _1}t_{lens})}{t_{lens}}\right) ^2 \end{aligned}$$

**Figure 2:**
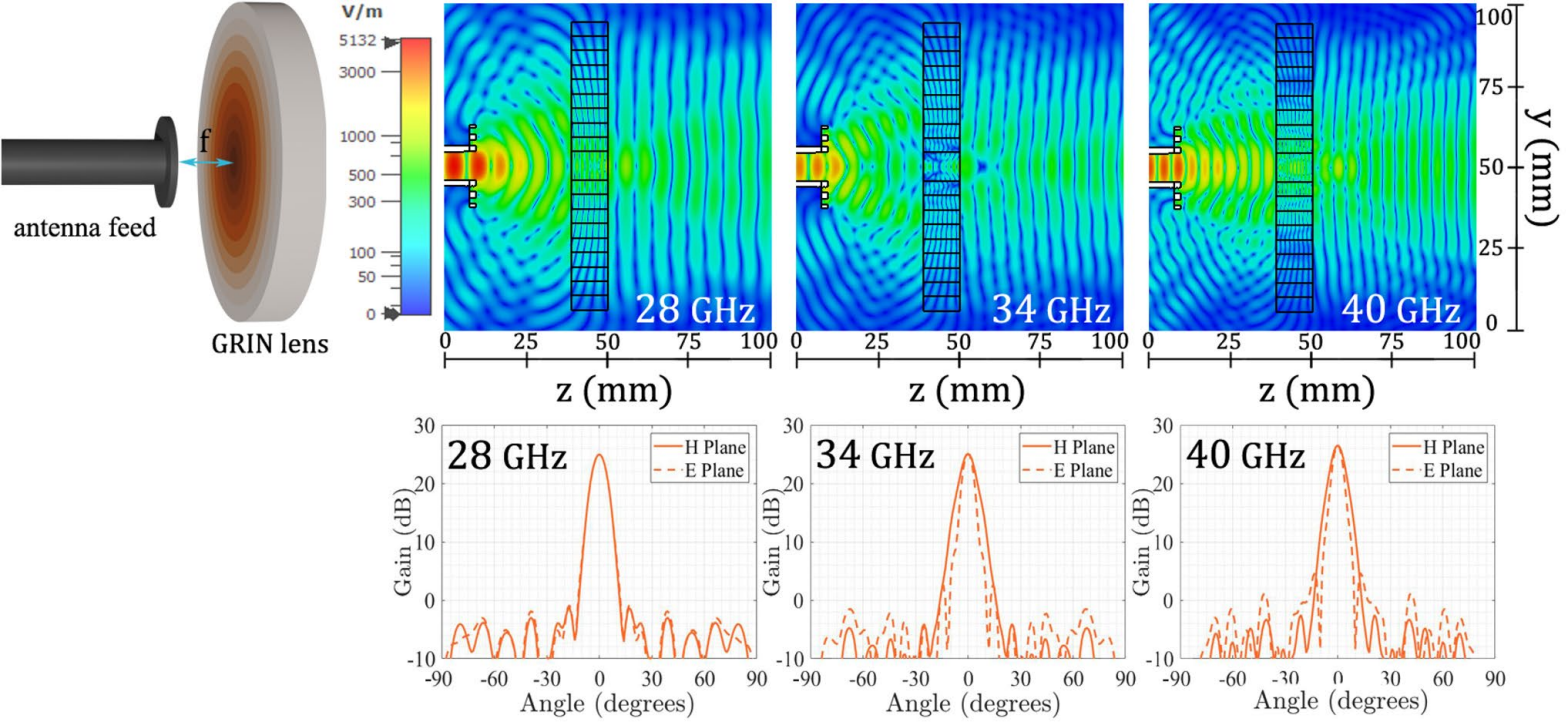
Simulated results of the 3D-printed flat lens at 27 GHz, 34 GHz and 40 GHz. Top: Electric field magnitude. Bottom: Gain radiation patterns.

Once the theoretical design is done, we proceed to simulate the lens using the full-wave simulation software CST Microwave Studio^29^, using a circular corrugated open waveguide feed positioned at the focal point ’*f*’ with a maximum gain of $10 \pm 0.5$ dB and a half-power beamwidth of $57^{\circ } \pm 4^{\circ }$ on both planes for the whole Ka-band. To assess the behavior of the designed lens, the resulting simulated electric field is shown in Fig. 2. The field is evaluated at three different frequencies over the band of interest, at three different relative positions inside the band, being the chosen frequencies 28 GHz, 34 GHz and 40 GHz. Simulation results show that the designed lens exhibits a good behavior in terms of transforming the incident wavefront into a plane wave, by keeping low back-scatter characteristics. To confirm the lens focusing characteristics, in Fig. 2 the gain radiation pattern in both planes (E-plane and H-plane) for the three same frequencies in the band of interest is also presented. The simulated results show that the designed lens keeps a good characteristic in terms of focusing on the analyzed frequencies, by having a maximum gain of around 25 dBi in the assessed frequencies.

**Figure 3:**
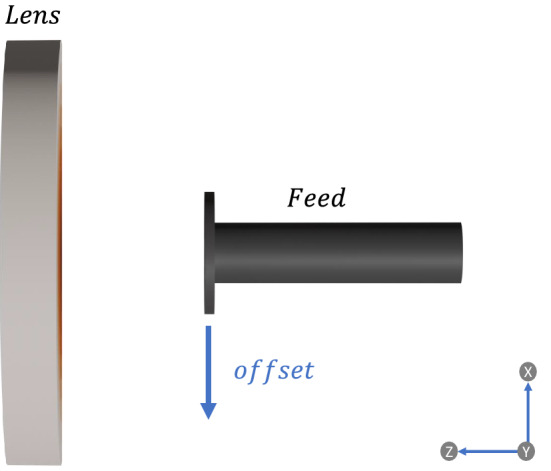
Side view of lens and feed with the corresponding the direction of feed offset.

**Figure 4:**
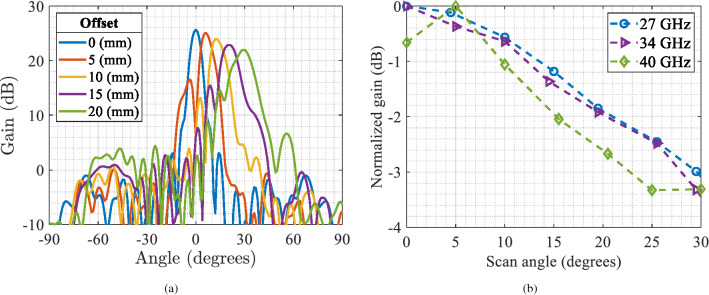
Simulation results of the lens behavior with different feed offsets. (**a**) Gain radiation pattern at 34 GHz. (**b**) Normalized gain as a function of the scanning angle at 27, 34 and 40 GHz.

Another interesting parameter to be evaluated is the beam-scanning capability of the lens, as its a feature required in many of the new communication systems and applications. Lens antennas can provide beam-scanning by adjusting or shifting the feed antenna, the same way as it is done when using reflectors. To notice that this shift was not done following the focal arc of the lens due to practical constraints in these kind of implementations. For the designed lens, we will use the previously used feed, and shift it an offset distance, as described in Fig. 3. Figure 4 shows the simulated radiation pattern obtained with five different feed offset positions and the reduction of the maximum gain as a function of the scanning angle. From the shown simulation results, we can observe that by moving laterally the feed, the lens can scan up to ± 30$^\circ$ with a maximum loss in the maximum gain of approximately 3 dB. However, we can expect better results if we shift the feed following the focal arc of the lens. It is important to say that the normalized gain obtained with a scan angle of 5$^{\circ }$ at 40 GHz (Fig. 4b) present higher value than the non-shifted result, due to the chromatic aberration phenomenon present in the lens, which produces a slight focus shift for frequencies far from the design frequency. However this difference is no larger than 0.6 dB.

**Figure 5:**
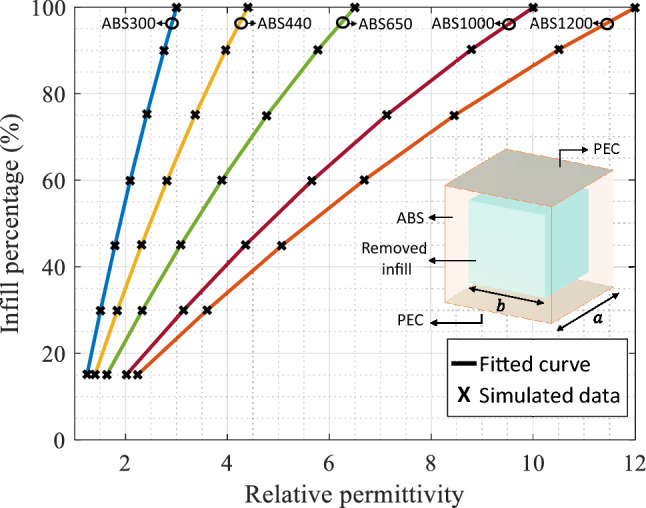
Relative permittivity as function of the infill percentage of each used filament. The inset display the configuration and parameters of the simulated unit cell.

Once the lens is designed and validated through simulation, we can proceed to its implementation. For the lens fabrication, it is necessary to manufacture each ring with its specific relative permittivity value obtained in the design process. For that, the lens is constructed using 3D-printing additive manufacturing process, using PREPERM dielectric filaments with different relative permittivity values^30^. To obtain the values that are not commercially available, we vary the infill percentage of each section to reduce the nominal relative permittivity of the filament. The needed infill percentage is obtained by calculating the dispersion diagram of a cubic unit cell, consisting of a parallel plate filled with a dielectric slab with different infill percentages, and then calculating the relative permittivity value of each case, obtaining the curves shown in the Fig. 5. The unit cell, shown in the inset of this figure, has a lateral dimensions of $a=\lambda /6$ and the *b* parameter depends on the simulated infill percentage, as done in previous works in lens designs^27^. To notice that as the unit cell is a sub-lambda structure, it properly represents the printing grid generated by the 3D-printer^19,27^. In Table 2 the infill percentages for each ring section with the corresponding filament are included.

**Table 2:** Ring relative permittivity ($\varepsilon _{r_n}$) section with the corresponding filament and infill.

Ring perm. value ($\varepsilon _{r_n}$)	Filament	Infill (%)
$\varepsilon _{r1}=12$	ABS1200 $\varepsilon _r=12$	100
$\varepsilon _{r2}=11.54$	ABS1200 $\varepsilon _r=12$	97.1
$\varepsilon _{r3}=10.78$	ABS1200 $\varepsilon _r=12$	92.1
$\varepsilon _{r4}=9.73$	ABS1000 $\varepsilon _r=10$	97.8
$\varepsilon _{r5}=8.49$	ABS1000 $\varepsilon _r=10$	87.7
$\varepsilon _{r6}=7.15$	ABS1000 $\varepsilon _r=10$	75.3
$\varepsilon _{r7}=5.79$	ABS650 $\varepsilon _r=6.5$	90.4
$\varepsilon _{r8}=4.48$	ABS650 $\varepsilon _r=6.5$	70.2
$\varepsilon _{r9}=3.28$	ABS440 $\varepsilon _r=4.4$	72.7
$\varepsilon _{r10}=2.22$	ABS300 $\varepsilon _r=3$	65.9

One thing to consider before the lens implementation is the large difference in permittivity at the air-lens transition, especially for the central rings, which can produce significant reflections. To reduce this effect, typically, matching layers are used^31,32^ . The parameters that describe each matching layer are the height *h* and its relative permittivity, whose values depend on the adjacent medias, as defined by Eq. (2). As there are two regions where we can have the air-dielectric contrasts, we will assess three cases for the matching layer implementation: air-lens contrast from the impinging wave side of the lens (namely PRE-ML case), lens ring-air transition at the outside of the lens (namely POST-ML case) and adding the matching layer as a combination of both cases (namely PRE&POST-ML case).

2 $$\begin{aligned} \begin{aligned}{}&\varepsilon _{r_{ml_{n}}}=\sqrt{\varepsilon _{r_0}\varepsilon _{r_n}} \\&\quad h_n=\frac{\lambda _0}{4\sqrt{\varepsilon _{r_{ml_{n}}}}} \end{aligned} \end{aligned}$$

**Figure 6:**
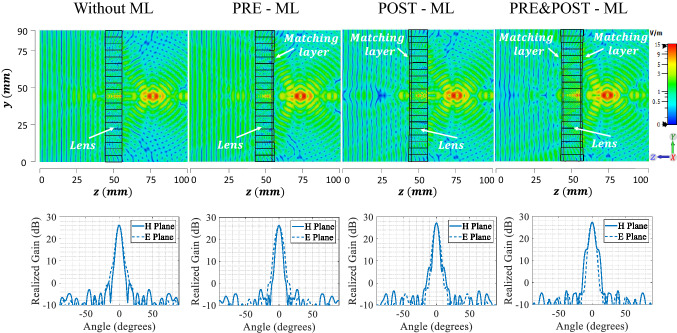
Simulated results of the 3D-printed flat lens at 34 GHz without matching layer, with matching layer before the lens (PRE-ML), with matching layer after the lens (POST-ML) and in both sides of the lens (PRE&POST-ML). Top: Electric field magnitude. Bottom: Gain radiation patterns.

In order to assess the effect of the different matching layer configurations on the lens characteristics, we simulate an impinging plane wave in the lens surface, moving in the $-z$ direction, as shown in Fig. 6. First, from the the simulated electric field results, we can see that adding a matching layer will slightly shift the focal point of the lens, with the larger shift exhibited for the PRE&POST-ML case of around 3.5 mm. Second, we evaluate the resulting gain radiation patterns using the matching layer. For that, we use the same corrugated feed structure, now placed at the new focal points obtained when adding the matching layers on the different study cases. The simulated gain results are presented at the bottom of Fig. 6, where we can see that the higher value of maximum realized gain is obtained for the PRE&POST-ML case, that is inline with the electric field magnitude simulations results.

Although adding a matching layer on both sides of the lens present the better simulations results, its a difficult topology to implement, due to manufacturing difficulties presented by a structure with both sides not flat. For example, as one of the matching layers will be on the bottom-side of the printing bed, it will need support layers that have to be removed. In addition, the fact of having different heights in contact with that support structure, will make impossible to precisely manufacture the lens in only one piece. Therefore, the option that will be manufactured in this work will be the one with the matching layer placed after the lens, which is the second best option when looking to the maximum gain.

**Table 3:** Relative permittivity and height of the matching layer rings with the filaments and infill used for manufacture.

Layer $\varepsilon _{r_{ml_n}}$	Height ($h_n$)	Filament	Infill (%)
$\varepsilon _{r_{ml_1}}=3.46$	1.19	ABS650 $\varepsilon _r=6.5$	52.2
$\varepsilon _{r_{ml_2}}=3.40$	1.20	ABS650 $\varepsilon _r=6.5$	51.0
$\varepsilon _{r_{ml_3}}=3.28$	1.22	ABS650 $\varepsilon _r=6.5$	48.8
$\varepsilon _{r_{ml_4}}=3.12$	1.25	ABS650 $\varepsilon _r=6.5$	45.8
$\varepsilon _{r_{ml_5}}=2.91$	1.29	ABS300 $\varepsilon _r=3$	96.3
$\varepsilon _{r_{ml_6}}=2.67$	1.35	ABS300 $\varepsilon _r=3$	86.4
$\varepsilon _{r_{ml_7}}=2.41$	1.42	ABS300 $\varepsilon _r=3$	74.9
$\varepsilon _{r_{ml_8}}=2.12$	1.52	ABS300 $\varepsilon _r=3$	61.4
$\varepsilon _{r_{ml_9}}=1.81$	1.64	ABS300 $\varepsilon _r=3$	45.9
$\varepsilon _{r_{ml_10}}=1.49$	1.81	ABS300 $\varepsilon _r=3$	28.9

**Figure 7:**
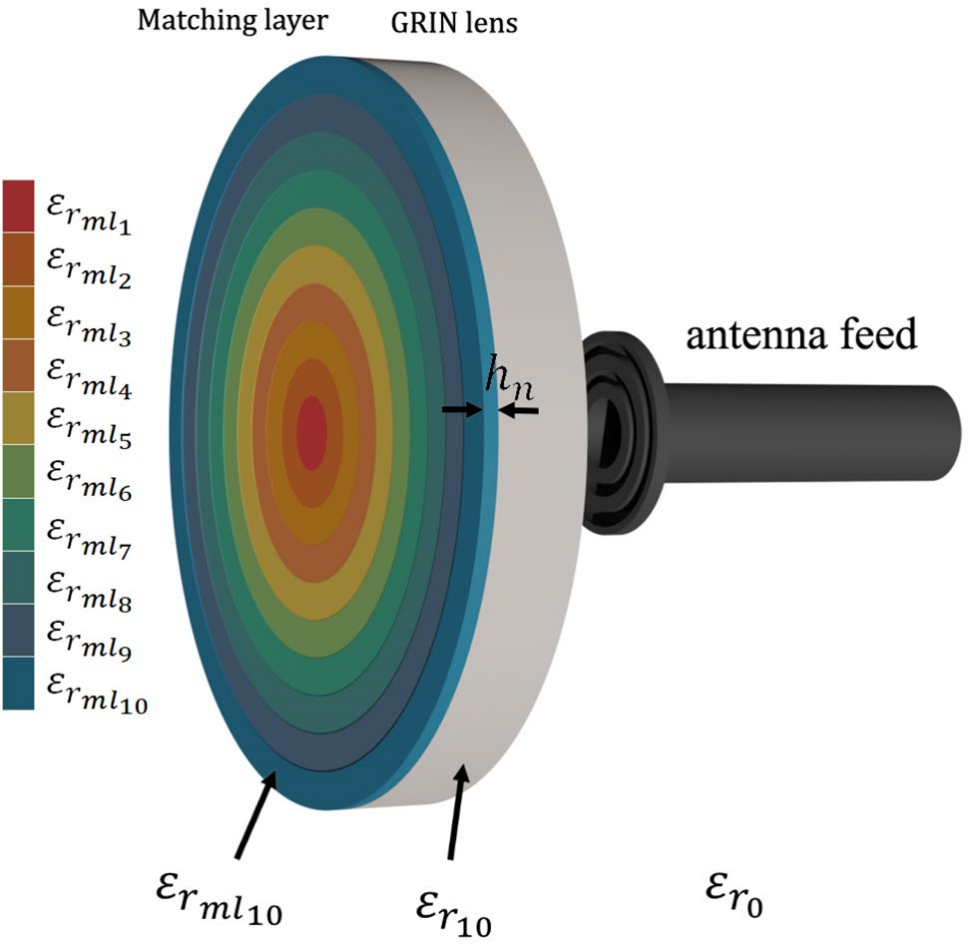
Model of the planar lens antenna with matching layer for each ring, fed by a corrugated circular open waveguide.

The height and permittivity value for each ring of the matching layer are shown in Table 3 and in Fig. 7 the lens antenna with the matching layers is presented. For the fabrication process, the same technique to achieve the different permittivity values applied in the lens rings is used. Table 3 shows the filaments used and their respective infill percentages for the each section.

**Figure 8:**
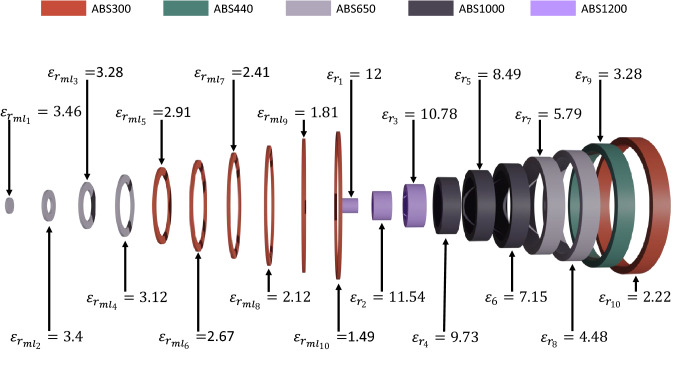
Exploded view of the lens and matching layer with the equivalent permittivity value required for each section and the filaments that will be used for each section.

**Figure 9:**
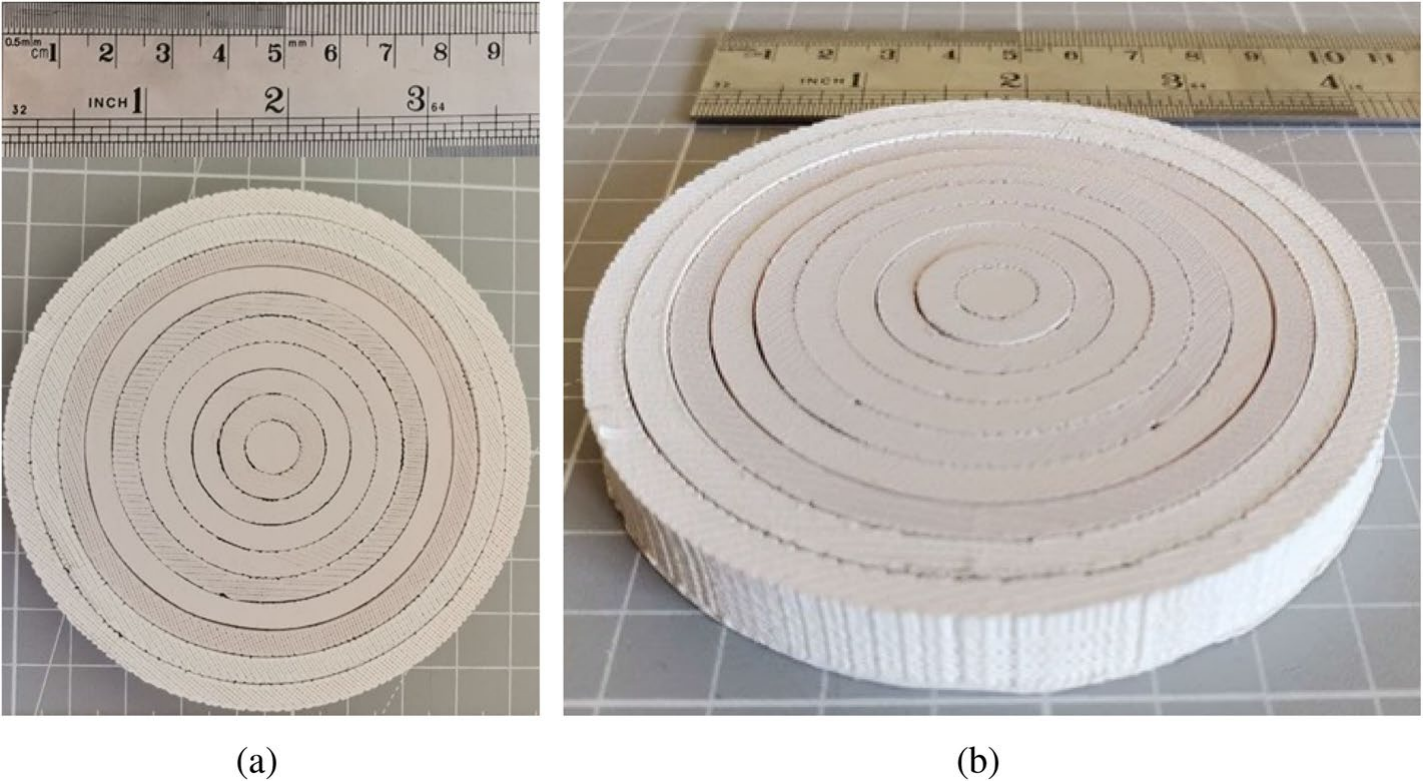
Photo of the manufactured 3D-printed planar lens antenna. (**a**) Top view. (**b**) Orthogonal view.

**Figure 10:**
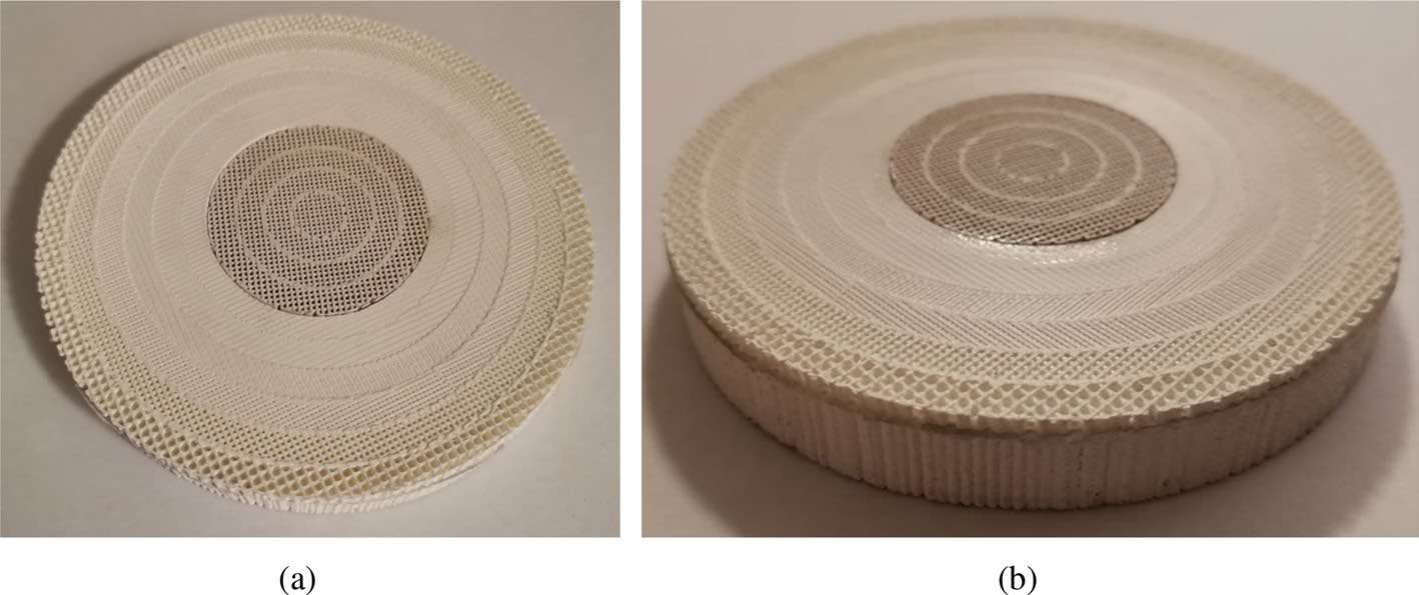
Photo of the manufactured matching layer and lens antenna. (**a**) Top view of the matching layer. (**b**) Lens antenna with matching layer.

The lens and the matching layers have been manufactured using a custom low-cost 3D-printer from Ocular3D^33^. For the lens, we use five different filaments whilst for the matching layers only two filaments are used, as shown Fig. 8 through the color coding. The lens and matching layer have been printed separately. For the lens, each ring has been printed individually and then glued together as shown in the Fig. 9, while for the matching layer only two pieces were made and glued (one for each used material), shown in Fig. 10. In Fig. 8 are shown an exploded view of the ten rings of the planar lens (right) and the ten rings of the matching layer (left), with their corresponding relative permittivity value and the filament used for their manufacture. The manufactured matching layer and the lens antenna were glued together, obtaining the prototype shown in Fig. 10b.

## Measurement results

**Figure 11:**
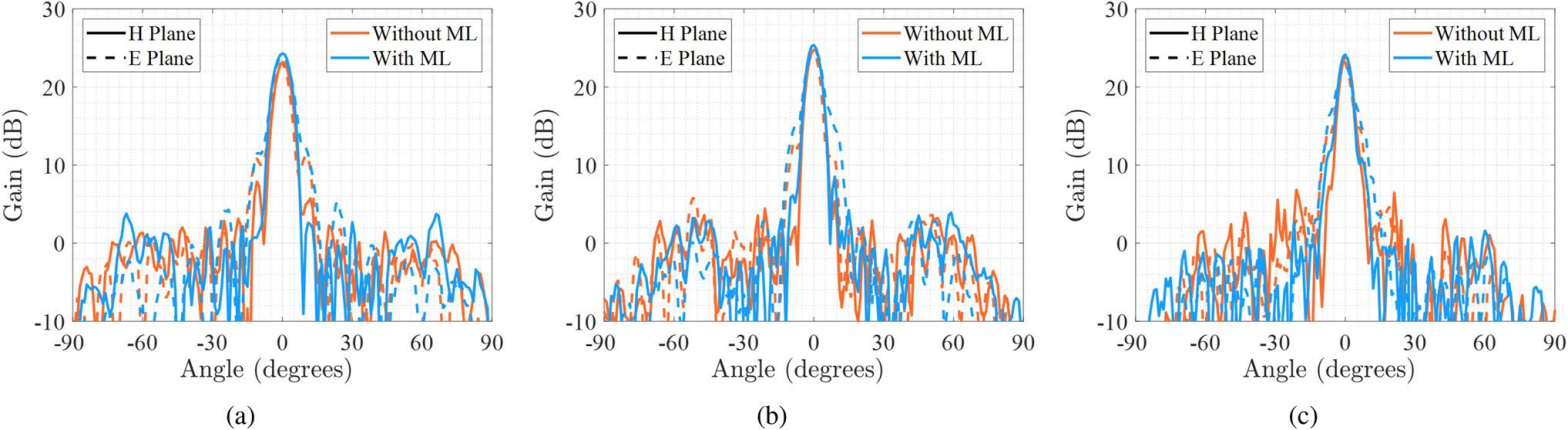
Measured E-plane and H-plane gain radiation pattern of the 3D-printed lens with and without matching layer at three frequencies in the Ka-band. (**a**) 28 GHz. (**b**) 34 GHz. (**c**) 40 GHz.

**Figure 12:**
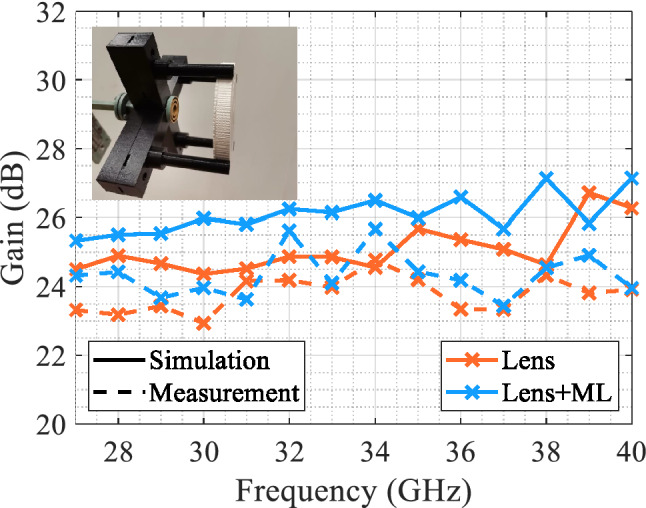
Maximum gain in Ka-band of the planar dielectric lens with and without matching layer. The inset displays a photo of the measurement setup and the lens with matching layer.

To validate the constructed lens, a wideband measurement of the gain radiation pattern is done in an anechoic chamber in the band of interest. The measurement setup consists of the same feed antenna used in simulations (i.e. maximum gain of $10 \pm 0.5$ dB, half-power beamwidth of $57 \pm 4^{\circ }$ on both planes for the whole Ka-band), with a PLA 3D-printed support structure that keeps the lens perpendicular to the feed axis. Figure 11 shows the gain radiation pattern of the lens with and without matching layer at 28 GHz, 34 GHz and 40 GHz. We can see that the behavior of the lens without the matching layer is similar to the results obtained by simulation, keeping low sidelobe levels and a similar maximum gain is measured. The measurements with the matching layer show an improvement in the lens behavior, obtaining higher levels of maximum gains but with a slight increment in the sidelobe levels. In Fig. 12, the maximum gain with and without the matching layer, simulated and measured in the Ka band is shown. The improvement in the maximum gain when using the matching layer in measurements is not as good as the obtained in simulations for the whole Ka-band. This can be due to the manufacturing accuracy constraints of the 3D printer, which for some sections were 0.2 mm in height, being comparable to the differences in heights required for different rings of the matching layer.

Finally, we can compare this lens solution with other 3D-printed planar lenses implemented in mm-wave frequency band. In Table 4 are shown different lens implementations compared in terms of its maximum gain and sidelobe levels (SLL), for the simulated and measured versions. From the table we can see that the presented lens has a better performance in both analyzed parameters, with a wide operational bandwidth. It is important to say that the lenses analyzed are also flat, in order to make a better comparison.

**Table 4:** Comparison of the presented lens with other planar lenses operating in millimeter wavebands present in the literature.

References	Relative bandwidth (%)	Meas. frequency (GHz)	Max. gain (dB)		SLL (dB)	
			Sim.	Meas.	Sim.	Meas.
Presented work	42.4	34	26.5	25.7	− 20	− 17.7
^34^	15.4	26	22.2	–	− 15.2	–
^14^	40	15	22	20	− 16	− 13.8
^35^	54.1	18.5	14.4	13.8	− 10.4	− 13.3
^36^	24.8	60	19	18.3	–	− 18
^37^	6.7	30	22.7	22	− 15.7	− 18
^22^	38.8	34	24	24	− 10	− 13

## Conclusions

This article presents the design and construction of a wideband cost-effective 3D-printed flat lens antenna for Ka band. The design includes also a matching layer that was added to the original lens design. Good agreement between measurement results and simulations is observed. The resulting lens is fully dielectric, low-cost, fully 3D-printed and planar, with high gain characteristics in the entire Ka-band, making it suitable for the new communication systems, such as 5G technologies.
